# The impact of stimulus configuration on visual short‐term memory decline in normal aging and mild cognitive impairment

**DOI:** 10.1002/brb3.3113

**Published:** 2023-06-08

**Authors:** Raju P. Sapkota, Ian van der Linde, Iris Q. Grunwald, Tirthalal Upadhyaya, Nirmal Lamichhane, Shahina Pardhan

**Affiliations:** ^1^ Vision & Eye Research Institute (VERI), School of Medicine, Faculty of Health, Education, Medicine and Social Care Anglia Ruskin University Cambridge UK; ^2^ School of Computing and Information Science Anglia Ruskin University Cambridge UK; ^3^ Imaging Science and Technology, School of Medicine University of Dundee Dundee UK; ^4^ Department of Medicine Gandaki Medical College Teaching Hospital Pokhara Nepal; ^5^ Diabetes, Thyroid and Endocrine Care Center Pokhara Nepal; ^6^ Department of Psychiatry Gandaki Medical College Teaching Hospital Pokhara Nepal; ^7^ BG Hospital and Research Center Pokhara Nepal

**Keywords:** aging, amnestic‐mild cognitive impairment, object configuration, spatial configuration, visual short‐term memory

## Abstract

**Introduction:**

When we memorize simultaneous items, we not only store information about specific items and/or their locations but also how items are related to each other. Such relational information can be parsed into spatial (spatial configuration) and identity (object configuration) components. Both these configurations are found to support performance during a visual short‐term memory (VSTM) task in young adults. How the VSTM performance of older adults is influenced by object/spatial configuration is less understood, which this study investigated.

**Methods:**

Twenty‐nine young adults, 29 normally aging older adults, and 20 older adults with mild cognitive impairment (MCI) completed two yes–no memory‐recognition experiments for four simultaneously presented items (2.5 s). Test display items were presented either at the same locations as the memory items (Experiment 1) or were globally shifted (Experiment 2). One of the test display items (target) was highlighted with a square box; participants indicated whether this item was shown in the preceding memory display. Both experiments comprised four conditions where nontarget items changed as follows: (i) nontarget items remained the same; (ii) nontarget items were replaced by new items; (iii) nontarget items switched locations; (iv) nontarget items were replaced by square boxes.

**Results:**

Performance (% correct) in both older groups was significantly reduced than young adults in both experiments and each condition. For the MCI adults, significantly reduced performance (vs. normal older adults) was found only for Experiment 1.

**Conclusion:**

VSTM for simultaneous items declines significantly in normal aging; the decline is not influenced differently by spatial/object configuration change. The ability of VSTM to differentiate MCI from normal cognitive aging is apparent only where the spatial configuration of stimuli is retained at original locations. Findings are discussed in terms of the reduced ability to inhibit irrelevant items and location priming (by repetition) deficits.

## Graphical abstract

We examined the impact of stimulus configuration on visual short‐term memory decline in cognitively normal older adults and older adults with mild cognitive impairment who are more at risk of developing dementia in the future. Retaining the stimulus configuration at the original location (but not at globally shifted locations) benefited task performance in cognitively normal older adults compared to older adults with mild cognitive impairment. We discuss findings in terms of the reduced ability of older adults to inhibit irrelevant items and location priming deficits.

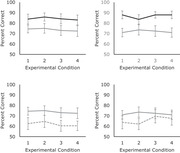

## 1 INTRODUCTION

Visual short‐term memory (VSTM), a transient memory system (lasting up to a few seconds) plays a vital role in supporting ongoing cognitive tasks (Phillips, 1974) and has been found to decline with the age (Borella et al., 2008; Chalfonte & Johnson, 1996; De Beni & Palladino, 2004; Fiore et al., 2012; Hasher & Zacks, 1988; Salthouse, 1990; Sapkota et al., 2015, 2020). Brain areas such as the prefrontal cortex and parts of medial temporal lobe (e.g., perirhinal cortex and hippocampus) that are implicated during a VSTM task are found to deteriorate with aging (Emery et al., 2008; Hampel et al., 2008; Reuter‐Lorenz & Cappell, 2008; Turner & Spreng, 2012).

Various postulations have been put forward to explain why the VSTM performance declines with normal aging. Salthouse (1990) suggested it to be due to a generalized deterioration in overall cognitive/executive functioning. According to Naveh‐Benjamin (2000), older adults are less able to associate different visual features of the same object (e.g., color and location) to form an integrated perceptual unit thereby leading to inferior VSTM performance compared to younger adults. Others propose that age‐related decline in VSTM performance is due to the reduced ability of older adults in inhibiting (or filtering) irrelevant visual information (Borella et al., 2008; De Beni & Palladino, 2004; Fiore et al., 2012; Gutchess & Boduroglu, 2019; Hasher & Zacks, 1988; Zacks & Hasher, 1994), or as a result of a greater decline in attentional control (Godefroy et al., 2010).

A commonly employed paradigm to study VSTM in the laboratory is the change detection paradigm, in which two multi‐item visual displays are shown one after another separated by a brief interstimulus interval, ISI (of up to a few seconds). Participants are required to indicate if the second (test) display changed visually from the first (memory) one (Luck & Vogel, 1997; Phillips, 1974; Wilken & Ma, 2004). In doing so, participants are found to not only store object and location information of individual items in VSTM but also the information about how those items are related to each other to form a specific layout (Brady & Alvarez, 2011; Jiang et al., 2000; Papenmeier et al., 2012; Timm & Papenmeier, 2019). Such relational information can be divided into spatial (spatial configuration) and identity (object configuration) components and have been found to serve as significant memory cues during a VSTM task in young adults (Jiang et al., 2000). Jiang et al. (2000) found that when stimulus configuration was preserved between memory and test displays, memory‐recognition performance for an item was significantly greater even where an absolute positional shift had been applied to the stimulus array compared to conditions where the configuration was not available (i.e., when memory targets were presented singly in the test display). Hence, the spatial configuration acts as an important source of memory retrieval.

The brain areas responsible for representing stimulus configuration in VSTM primarily involve perirhinal cortex, hippocampus, and prefrontal cortex and the interplay between them (Olson et al., 2006; Rao et al., 1997; van Asselen et al., 2005). Perirhinal lesions have been found to impair memory recognition for identity configuration (Bartko et al., 2007), whereas the hippocampal lesions in mild cognitive impairment (MCI) or early dementia impair memory recognition for spatial configuration (Kesner, 2013). Although the brain areas representing stimulus configuration in VSTM (mentioned above) are known to deteriorate with normal aging and in early dementia, little is known about how age‐related decline in VSTM is influenced by changes in stimulus configuration. Furthermore, previous studies have not investigated this question in people with amnestic MCI, a group known to be at higher risk of dementia (Petersen et al., 1999), despite the fact that spatial processing of items is significantly impaired in these people (Kessels et al., 2010). These two questions were investigated in the current study in two yes–no memory‐recognition experiments for simultaneously presented items in young healthy adults, cognitively normal older adults, and cognitively impaired older adults (amnestic MCI adults). In Experiment 1, the spatial configuration of stimuli was maintained at the original locations, and in Experiment 2, the spatial configuration was maintained at globally shifted locations. Based on the gaps in the literature, the following hypotheses and expected outcomes were tested:

1. Cognitively normal older participants perform significantly worse than young participants in a VSTM task for simultaneously displayed items; this performance difference between the two groups is not influenced by changes in the spatial configuration of stimuli.
2. Participants with MCI are less able to apply the same‐location configurational cues during a VSTM task compared to cognitively normal older adults of similar age.

Our overall aim was to identify if (or which of the) configuration‐based cues of VSTM are associated with normal cognitive aging and in amnestic‐MCI and show potential to be used as a marker for differentiating normal cognitive aging from early (preclinical) dementia.

## 2 EXPERIMENT 1

This experiment examined how normal aging and MCI influenced VSTM performance for simultaneously presented items in which spatial configuration of stimuli was maintained at the same location between memory and test display, but object configuration of nontarget items was varied.

### 2.1 Method

#### 2.1.1 Participants

Twenty‐nine healthy young adults (M = 24.7 years, SD = 3.6, number of females = 15), 29 cognitively normal older adults (M = 64.6 years, SD = 4.6, number of females = 15), and 20 older adults with amnestic MCI (M *=* 66.0 years, SD = 4.9, number of females = 10) who were fluent in English (second language) completed two VSTM experiments. All participants had normal or corrected‐to‐normal vision and no hearing impairment. Cognitively normal older adults and young healthy adults scored more than 88 points (out of 100) on the Addenbrooke's Cognitive Examination‐III test (Hsieh et al., 2013). Participants with amnestic MCI were diagnosed by author NL, who is a clinical neuropsychiatrist; ACE‐III scores of these participants ranged from 75 to 88 with an overall score for memory domain of <24 points, which is the ACE‐III criteria for identifying amnestic MCI (Hsieh et al., 2013). ACE‐III total scores and the scores for individual subscales are provided in the Supplementary Material 1.

The proposed sample size exceeds the number (*n* = 10 MCI subjects) used in another study that used a similar methodological approach to study the effect of MCI in VSTM for sequentially presented items (Sapkota et al., 2017).

All participants provided informed consent before taking part in the study. Participants were treated in accordance with the applicable ethical guidelines that followed the tenets of the Helsinki Declaration. Ethical approval was obtained from the Institutional Review Board of Gandaki Medical College and Teaching Hospital, Pokhara, Nepal.

#### 2.1.2 Stimuli and apparatus

**FIGURE 1:**
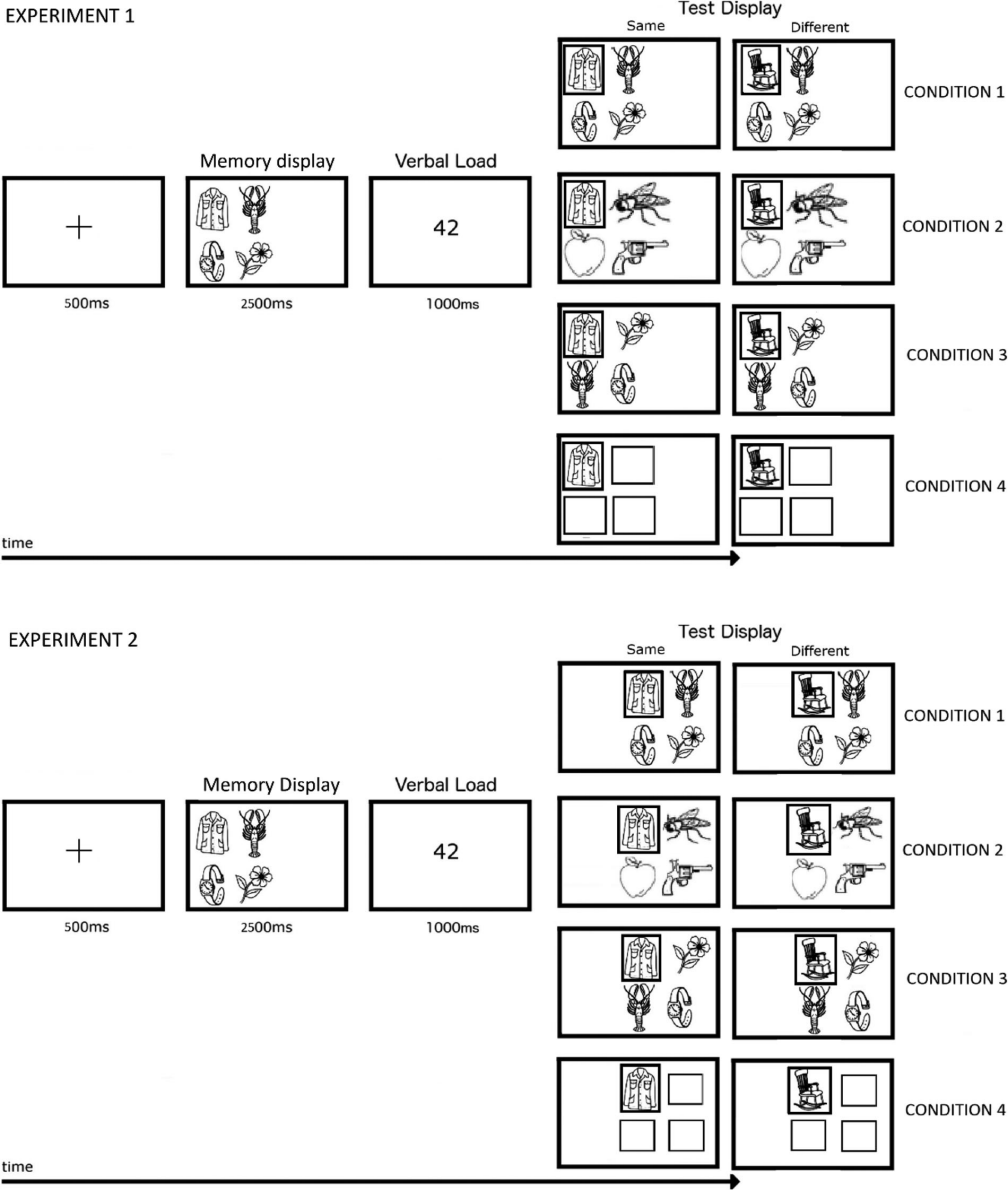
Schematic representation (not to‐the‐scale) of the test procedures used in conditions 1–4 (which differed in terms of how nontarget items changed between memory and test displays) of Experiments 1 and 2. Condition 1 nontarget items remained unchanged; condition 2 nontarget items were replaced by new items; condition 3 nontarget items switched locations; condition 4 nontarget items were replaced by empty square boxes. Items in the test display were presented at the same locations used to present items in the memory display in Experiment 1. In Experiment 2, items in the test display were globally shifted to new locations but maintaining the same relative distance between stimuli in the memory display.

Stimuli comprised 180 line drawings of real‐world objects, such as animals, birds, fruits, vegetables, and toys taken from Snodgrass and Vanderwart (1980), each subtending 2.5° of visual angle at a testing distance of 57 cm. Example stimuli are shown in Figure 1. Nameable stimuli were used (rather than non‐nameable novel items) for ecological validity.

Stimuli were displayed using a Sony laptop computer (Sony Corporation, Model: PCG‐71313 M, Japan) with a screen resolution of 1366 × 768 pixels and a refresh rate of 60 Hz. The viewing distance was set to be approximately 57 cm. The ambient light was held constant across trials and between participants.

Stimulus presentation was controlled by MATLAB (Mathworks, Natick, MA, USA) with the Psychtoolbox/Video Toolbox extensions (Brainard, 1997; Pelli, 1997). The stimulus background was set to mid‐gray.

#### 2.1.3 Procedure

All eligible participants completed two VSTM experiments (see later). Participants wore their glasses if needed for the testing distance (57 cm approximately). Test procedures were preceded by a stimulus learning routine in which all 180 stimuli were displayed sequentially in random order. All participants were able to recognize and name all the stimuli correctly before taking part in experimental trials.

A schematic representation of the test procedures used in Experiments 1 and 2 are shown in Figure 1.

Each trial began with the presentation of a 2.5° fixation cross displayed at the screen center for 500 ms. This ensured that all participants fixated upon a common screen position prior to the presentation of a memory display (also referred to as the study display) in which four items were shown simultaneously in an imaginary square configuration (7.5° × 7.5°) for 2.5 s; minimum edge‐to‐edge separation between any two adjacent items was 2.5°. It has been previously suggested that a display duration of as short as 500 ms is sufficient to encode simultaneously viewed four items in VSTM (Luck & Vogel, 1997).

The (x,y) coordinates of the stimuli locations in the memory display varied randomly between trials. We used a set of four memory items to align with the commonly cited three to four‐item capacity of VSTM (Irwin & Zelinsky, 2002; Luck & Vogel, 1997; Pashler, 1988). Next, a two‐digit number was shown at the display screen center for 1 s, which participants had to read aloud as it appeared (to promote *verbal suppression*, Baddeley, 1986; Todd & Marois, 2004). This was followed by the presentation of a test display in which the items were shown at the original locations used in the memory display. A square box was used to cue the target item in the test display, and participants were required to indicate whether it was one of the four items shown in the memory display, that is, the yes–no memory‐recognition paradigm. As the target item was cued only in the test display, participants did not know beforehand which of the four memory display items was going to be subsequently tested, that is, they were required to remember all the items from the memory display to produce a correct response.

In signal present trials (in which participants should respond *yes*), the target item was one of the four items presented in the preceding memory display, whereas in the signal absent trials (in which participants should respond *no*), a new item was cued as the target item in the test display. Signal present and signal absent trials occurred equally often and were randomly interleaved. Participants registered their responses by clicking a computer mouse (left key for a *yes* response, right key for a *no* response). Participants were instructed to focus on the accuracy than on speed, that is, it was a non‐speeded task, and reaction time was therefore not examined. The next trial commenced immediately after a response was submitted. As the task was a non‐speeded, the timing between trials was not strictly controlled.

There were four conditions that differed in terms of how the configuration of the nontarget items varied between the memory and the test display (Figure 1): (i) condition 1: nontarget items remained the same; (ii) condition 2: nontarget items were replaced by new items (i.e., they were different images than those in the preceding memory set); (iii) condition 3: nontarget items were the same but they switched locations; (iv) condition 4: nontarget items were replaced by empty square boxes. Comparison between conditions enabled us to examine the following effects: (i) effect of using new object configuration (conditions 1 vs. 2), (ii) effect of updating object configuration at switched locations (conditions 1 vs. 3), and (iii) effect of the absence of object configuration (conditions 1 vs. 4).

Each participant completed three blocks of 16 trials resulting in 48 trials in total, with 12 trials per condition (mentioned above) across blocks. On pretesting on a small number of participants (not included in our current analysis), elderly participants, in particular, those with the MCI had reported that they could do above 50 trials reasonably easily without being overwhelmed by the task. A previous on sequential display study of VSTM in individuals with MCI reports data from 16 trials from each participant (Sapkota et al., 2017). All the four conditions were tested within each block with the trials randomly interleaved (four trials per condition per block) minimizing any test order effects. As trials of different conditions were randomly interleaved in each block, neither participant nor the data collector had any prior knowledge of which condition trial type was going to be tested next. There was a brief pause of 2 min between each block. Furthermore, participants could also rest as often and for as long as they wished by withholding their responses. No adverse events or side effects were experienced by any participant during testing.

It took approximately 18–20 min for each participant to complete the experiment, including the ACE‐III test. Moreover, each participant completed a practice block of 16 trials (that were not included in the analysis) to familiarize themselves with the test procedures before taking part in the actual experiment. A trained data collector under the supervision of experienced researchers (NL and TLU) collected data.

Data were categorized according to the signal detection theory measures (Stanislaw & Todorov, 1999). The task performance was analyzed in terms of % correct response instead of d‐prime since, as occasionally participants’ performance for signal present and or signal absent trials was 100% (yielding extreme values of hit rate = 1 and false alarm rate = 0). According to Stanislaw and Todorov (1999), if one rate has an extreme value and the other does not, d′ is indeterminate, and in such a situation, one possibility is to quantify sensitivity with nonparametric measures, such as A prime. Hence, the corrected measure of sensitivity (A prime‐ that ranges from 0 to 1) was calculated using previously used formulae (Mueller & Zhang, 2006; Pavan et al., 2021). Response/decision bias (β) was calculated using the formula provided in Zhang and Mueller (2005).

To examine the effect of normal cognitive aging on VSTM performance (primary outcome 1), data were analyzed using a two‐way mixed ANOVA with participant group (young healthy adults and cognitively normal older adults) as the between‐subjects factor and test conditions (1–4) as within‐subjects factors. Similarly, to examine the effect of amnestic MCI on VSTM performance (primary outcome 2), data were analyzed using a two‐way mixed ANOVA with participant group (cognitively normal older adults and amnestic MCI adults) as the between‐subjects factor and test conditions (1–4) as within‐subjects factors. Assumptions underlying the ANOVA test such as the normality of data and the independence of sample distribution were met. Bonferroni correction was applied for pairwise multiple comparisons to compensate for type I error rate inflation. Where the assumption of sphericity was violated (identified using Mauchly's test), degrees of freedom were adjusted using the Greenhouse–Geisser procedure. Effect sizes were measured in terms of Cohen's *d* or partial eta squared (*ηp* ^2^). A prime data were analyzed by using nonparametric test.

### 2.2 Results

The mean age of young participants was 24.7 years (SD = 3.6). The mean age of normally aging older participants was 64.6 years (SD = 4.6), and the mean age of older participants with MCI was 66.0 years (SD = 4.9). The mean age of the latter two groups did not differ significantly (*t*(47) = −1.01, *p* = 0.29, Cohen's d = 0.31). The participant groups did not differ significantly in terms of the number of years of education (*t*(47) = −0.8, *p* = 0.43, Cohen's d = 0.36).

#### 2.2.1 The effect of aging

Our data suggest that cognitively normal adults performed the task significantly less well than young healthy adults, but significantly better than MCI adults in the condition where the spatial configuration of stimuli was preserved at original locations. In addition, this was observed even when the object configuration of nontarget items was changed to new items switched between stimuli locations or absent during the test.

Because stimuli were presented at the same locations between the memory and the test displays in Experiment 1, it is not clear whether our findings were influenced by priming due to the same location being tested. To clarify this, Experiment 2 was run in which test items were presented at globally shifted locations. The original spatial configuration was retained but stimuli were presented at new locations to eliminate the effect of priming of stimuli location at test. It is possible that semantic priming arising from the presentation of conceptually related items (Jackendo, 1976) could have influenced memory performance in some trials. For example, presenting an image of a screwdriver might influence the decision about an image of a nail since both items belong to the same conceptual category of “carpenter's tools” (Snodgrass & Vanderwart, 1980). However, as the same stimuli set was used in both experiments, any semantic priming is likely to be similar for both groups of older participants in both experiments. On the other hand, location priming, which arises from the repeated use of the same locations between the memory and the test displays, would have been potentially advantageous in Experiment 1 but not in Experiment 2. According to a previous model (Nicoletti & Umilta, 1994), if memory display locations are reused in the display of test items, the spatial codes created during the memory display help to guide attention during the test display. MCI participants were reported to show greater visuospatial coding deficits during visual attention tasks compared to healthy controls (Zhang et al., 2015).

## 3 EXPERIMENT 2

Participants, stimuli and apparatus, procedures, and experimental conditions were the same as in Experiment 1 except items in the test display were shown at the globally shifted (at random) new locations (Figure 1). It took approximately 6–8 min for each participant to complete the experiment.

### 3.1 Results

#### 3.1.1 The effect of aging

**FIGURE 2:**
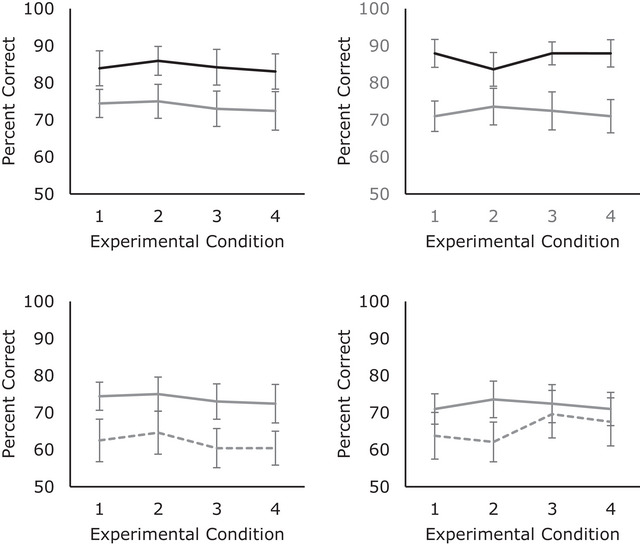
Left panel‐memory accuracy (mean % correct) response across experimental conditions 1–4 in Experiment 1. Above young adults (solid black line) versus cognitively normal older adults (solid gray line). Below cognitively normal older adults (solid gray line) versus older adults with amnestic mild cognitive impairment (MCI) (dotted gray line). Error bars represent 95% confidence interval. Right panel: memory accuracy (mean % correct) response across experimental conditions 1–4 in Experiment 2. Above young adults (solid black line) versus cognitively normal older adults (solid gray line). Below cognitively normal older adults (solid gray line)) versus older adults with MCI (dotted gray line). Error bars represent 95% confidence interval.

Figure 2 (right‐hand panel‐above) shows how memory performance (mean % correct response) differed between cognitively normal older adults and young healthy adults across test conditions in Experiment 2. A significant main effect of the participant group was found in which cognitively normal older adults performed the task significantly less well than young healthy adults, *F*(1,56) *=* 53.63, *p* *<* .001, *η_p_* ^2^ = .49, and in each condition (*p ≤* .005). There was no significant interaction effect, *F*(3,168) = 1.51, *p =* .22, *η_p_* ^2^ = .03. These data suggest that the effect of normal cognitive aging on VSTM performance for simultaneously presented items is robust to change in the spatial configuration of stimuli from original to globally shifted locations.

**FIGURE 3:**
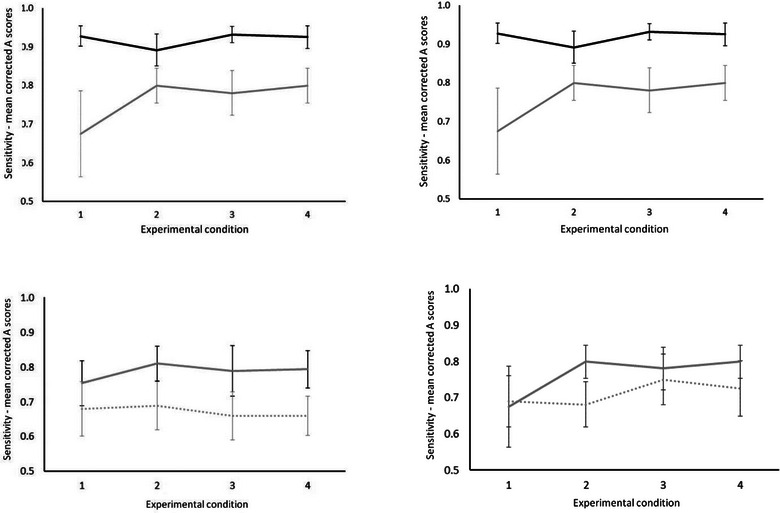
Left panel‐sensitivity (mean corrected A scores) across experimental conditions 1–4 in Experiment 1. Above young adults (solid black line) versus cognitively normal older adults (solid gray line). Below cognitively normal older adults (solid gray line) versus older adults with amnestic mild cognitive impairment (MCI) (dotted gray line). Error bars represent 95% confidence interval. Right panel‐ sensitivity (mean corrected A scores) across experimental conditions 1–4 in Experiment 2. Above young adults (solid black line) versus cognitively normal older adults (solid gray line). Below cognitively normal older adults (solid gray line) versus older adults with MCI (dotted gray line). Error bars represent 95% confidence interval.

Figure 3 (right‐hand panel‐above) shows how sensitivity (mean corrected A scores) differed between normally aging older adults and young healthy controls across test conditions in Experiment 2. Analysis of the A prime showed a significant difference between young and cognitively normal older adults, *χ* ^2^ (1) = 12.4, *p* < .001.

#### 3.1.2 The effect of MCI

Figure 2 (right‐hand panel‐below) shows how memory performance differed between older adults with amnestic MCI and cognitively normal older adults in each test condition of Experiment 2. Unlike in Experiment 1, the overall performance of MCI adults was not found to be significantly lower than cognitively normal older adults, *F*(1,47) = 1.47, *p* = .23, *η_p_^2^* = .03, and for any of the experimental conditions (*p* ≥ .08). The data suggest that while the effect of normal cognitive aging on VSTM performance is robust to the global shift in stimulus locations, the effect of amnestic MCI is not, that is, MCI adults performed the task without differing significantly from cognitively normal older adults at the globally shifted new locations. Figure 3 (right‐hand panel‐below) shows how sensitivity (mean corrected A scores) differed between normally aging older adults and older adults with MCI across test conditions in the Experiment 2. Analysis of the A scores showed a nonsignificant difference between the MCI and cognitively normal older adults, *χ* ^2^ (1) = 1.8, *p* = .26.

One may argue that our results may have been driven by spatial crowding effects since multiple stimuli were presented in the memory and the test display. To address this possibility, we ran a follow‐up condition in which only one item was presented in the test display. Similar patterns of results (to above) were observed,^1^ discounting this possibility. Moreover, it is worth highlighting that our stimuli were presented sufficiently far apart (edge‐to‐edge distance ≥2.5°) to minimize spatial crowding during memory encoding (Flom et al., 1963; Polat & Sagi, 1993).

We also ran a three‐way ANOVA with participant group (young healthy adults, cognitively normal older adults, and older adults with MCI) as the between‐subjects factor, and experiment type (Experiments 1 and 2) and test conditions (1–4) as within‐subjects factors, which revealed a nonsignificant main effect of experiment type, *F* (1,225) = 1.77, *p* = .19, *η_p_^2^* = .02, and nonsignificant interaction between experiment type, experimental condition, and participant groups, *F*(6,225) = 0.98, *p* = .44, *η_p_* ^2^ = .02. Decision biases for the three participant groups are provided in Supplementary Material 2. No statistically significant difference in decision bias between the participant groups was found, *F*(2,77) = 2.5, *p* = .09, *η_p_* ^2^ = .06. Moderate to good intertrial reliability was obtained for both, Experiments 1 and 2 with intertrial coefficients of ≥.5 as determined by the formula given in Fortin et al. (2008). Sensitivity analysis showed the most significant differences between the MCI and the normally aging older adults in test conditions 1 and 3 in Experiment 1 and condition 2 in Experiment 2.

## 4 DISCUSSION

This study advances the idea of why we forget items more easily from our VSTM as we become older or if we have early dementia. To our knowledge, this is the first study to examine VSTM performance for simultaneously presented items using object recognition (*yes–no*) memory experiments in three participant groups: young healthy adults, cognitively normal older adults, and older adults with amnestic MCI. Performance comparison between cognitively normal older adults and young healthy adults enabled us to examine the effect of normal aging, whereas the performance comparison between older adults with amnestic MCI and cognitively normal older adults enabled us to examine the effect of amnestic MCI. In Experiment 1, stimulus configuration was retained between memory and test displays at the same locations. In Experiment 2, stimuli were globally shifted to new locations between the memory and test displays, that is, the spatial configuration of stimuli was retained but at the new locations.

A significant effect of normal aging on VSTM performance was found in both experiments, suggesting that age‐related decline in VSTM performance is robust to changes in the spatial configuration of stimuli from original to the globally shifted new locations. Hasher and Zacks (Hasher & Zacks, 1988; Zacks & Hasher, 1994) suggested that VSTM performance decreases with the advancing age because of a deterioration of older adult's ability to inhibit irrelevant visual information; in our experiments, nontarget items. Our results add to this hypothesis by suggesting that the deterioration of older adult's ability to inhibit irrelevant memory representations is evident irrespective of whether the VSTM is tested by retaining spatial configuration of stimuli at original locations or at globally shifted new locations.

We also explored how VSTM performance for simultaneously presented items differed between cognitively normal older adults and older adults with amnestic MCI. Although amnestic MCI adults showed a significantly lower performance than cognitively normal older adults in Experiment 1, memory performance between these two groups did not differ significantly in Experiment 2 (although the observed effect size was relatively small). Our data suggest that reduced VSTM performance in MCI adults compared to normally aging older adults is observed only where the spatial configuration of stimuli is retained at original locations. We propose this to be due to the reduced ability of MCI adults to apply same‐location priming to benefit the task performance. The ability of VSTM to discriminate amnestic MCI from cognitively normal older adults is only apparent where the stimuli array remained at the same position, that is, in which same‐location priming could not be capitalized upon by the MCI adults. Previous studies on adults with MCI and Alzheimer's disease have shown that VSTM performance is reduced significantly under conditions in which priming for object‐location or color‐location is interfered (Parra et al., 2009; Sapkota et al., 2017).

An alternative explanation to the finding of significant difference between cognitively normal older adults and older adults with amnestic MCI in Experiment 1 but not in Experiment 2 may be that unlike in Experiment 1, both MCI and cognitively normal older adults found it similarly difficult to update globally shifted spatial locations in Experiment 2. It is also worth pointing out that the performance differences in Experiment 1 between condition 1 (where nontarget items were represented at original locations) and condition 3 (where nontarget items switched locations) were not significant within any participant group, suggesting that no benefit due to object‐location binding (if any) from nontarget items was found. However, previous studies have found object‐location binding for target items during a VSTM task (Chalfonte & Johnson, 1996; Hollingworth, 2006; Mitchell et al., 2000; Sapkota et al., 2020). It is possible that in our study, the distributed attention required for maintaining multiple items (target and nontarget) in VSTM during the ISI is reallocated to focus only on the cued target item in the test display, a mechanism driven possibly by the top‐down selective attention (Sun & Gordon, 2010). Consequently, this might have prevented/masked object‐location binding pertaining to nontarget items during the test.

One may argue that memory performance may decline anyway in older adults, that is, in both, with and without amnestic MCI. However, it should be noted that not all features of VSTM are influenced by aging or MCI. For example, Naveh‐Benjamin (2000) used a *yes–no* recognition task for sequentially presented words and nonwords found that memory for individual words did not differ significantly between young and elderly adults, but memory for word‐to‐nonword pairs differed significantly. Likewise, previous data show no significant performance difference between MCI adults and cognitively normal older adults during a VSTM recognition task for sequentially presented objects or locations, but a significant difference for object‐location binding (Sapkota et al., 2017).

One may suggest that both groups of elderly participants were similarly capable of utilizing semantic priming (if any) arising from the presentation of conceptually related items in both experiments, but location priming (arising from the repeated use of the same locations between the memory and the test displays) that could have favored cognitively normal older participants (vs. MCI) in Experiment 1 was absent in Experiment 2 which then resulted in a nonsignificant performance difference between the MCI and cognitively normal older participants. Training participants by repeatedly showing the study stimuli between the experimental blocks may enhance the influence of semantic priming on VSTM performance, which will be examined in our future study. We also did not consider modeling our data with one of the VSTM models because of the limited number of trials collected from each participant. We will investigate this in our future study on a larger number of participants.

To conclude, our data suggest that VSTM performance for simultaneously presented items declines significantly in normal aging; such a decline is robust to change in the spatial configuration of stimuli from original to the globally shifted to new locations. Change in object configuration does not influence VSTM performance decline in normal aging and amnestic MCI. Although the priming of stimulus configuration at the same location was not found to be a significant factor in explaining the age‐related decline in VSTM performance, it was found as a potential significant factor in explaining the memory decline in amnestic MCI adults (a group who are found to be at greater risk of developing dementia). Our findings would be useful in improving tests of VSTM aimed at identifying early cognitive decline or differentiating normal cognitive aging from early dementia (Sapkota et al., 2017). The findings also may be helpful in furthering our understanding of how people with normal and reduced cognitive abilities interact with the everyday visual environment where to‐be‐remembered items frequently change in their form and/or their spatial configuration.

## Funding information

This work was supported by internal funding from the Vision and Eye Research Institute (VERI), Anglia Ruskin University, Cambridge, UK.

## CONFLICTS OF INTEREST

All the authors have read the papers and have agreed to be listed as authors. We declare that the research was conducted in the absence of any commercial or financial relationships that could be construed as a potential conflict of interest.

### PEER REVIEW

The peer review history for this article is available at https://publons.com/publon/10.1002/brb3.3113.

## Supporting information

Supplementary material 1 ACE‐III total and the scores for individual cognitive subscales.
Supplementary material 2 Summary of decision biases for three groups of participants with results of post hoc comparisons between the individual groups.

## Data Availability

The data that support the findings of this study are available from the corresponding author upon reasonable request.

## NOTE

1 A sub‐analysis for each participant group showed that the mean % correct response was found to be significantly greater where the spatial configuration of stimuli was available (condition 1) at test than where it was not available (condition 4) in both experiments (*p <* .05), supporting the previous finding that spatial configuration facilitates VSTM task performance.
