# Supplementary material for: The impact of stimulus configuration on visual short‐term memory decline in normal aging and mild cognitive impairment
Source: Brain Behav. 2023 Jun 8;13(9):e3113. doi: 10.1002/brb3.3113 (PMC10498075; doi:10.1002/brb3.3113)
Supplement: Supplementary file 1 — Supplementary material 1 ACE‐III total and the scores for individual cognitive subscales. Supplementary material 2 Summary of decision biases for three groups of participants with results of post hoc comparisons between the individual groups. [file BRB3-13-e3113-s001.docx]

Supplementary material 1. ACE-III total and the scores for individual cognitive subscales

Supplementary material 2. Summary of decision biases for three groups of participants with results of post hoc comparisons between the individual groups
